# Accelerated Telomere Attrition Is Associated with Relative Household Income, Diet and Inflammation in the pSoBid Cohort

**DOI:** 10.1371/journal.pone.0022521

**Published:** 2011-07-27

**Authors:** Paul G. Shiels, Liane M. McGlynn, Alan MacIntyre, Paul C. D. Johnson, G. David Batty, Harry Burns, Jonathan Cavanagh, Kevin A. Deans, Ian Ford, Alex McConnachie, Agnes McGinty, Jennifer S. McLean, Keith Millar, Naveed Sattar, Carol Tannahill, Yoga N. Velupillai, Chris J. Packard

**Affiliations:** 1 Institute of Cancer Sciences, College of Medical, Veterinary and Life Sciences, University of Glasgow, Glasgow, Scotland; 2 Robertson Centre for Biostatistics, University of Glasgow, Glasgow, Scotland; 3 Medical Research Council Social and Public Health Sciences Unit, Glasgow, Scotland; 4 Clinical Epidemiology Group, Department of Epidemiology and Public Health, University College London, London, England; 5 Scottish Government, Edinburgh, Scotland; 6 Section of Psychological Medicine, University of Glasgow, Glasgow, Scotland; 7 Department of Clinical Biochemistry, NHS Greater Glasgow and Clyde, Glasgow Royal Infirmary, Glasgow, Scotland; 8 Department of Clinical Biochemistry, Aberdeen Royal Infirmary, Aberdeen, Scotland; 9 Glasgow Clinical Research Facility, Glasgow, Scotland; 10 Glasgow Centre for Population Health, Glasgow, Scotland; 11 Institute of Cardiovascular and Medical Sciences, College of Medical, Veterinary and Life Sciences, University of Glasgow, Glasgow, Scotland; 12 Epidemiology and Public Health Department, University College Cork, Cork, Ireland; Virginia Tech, United States of America

## Abstract

**Background:**

It has previously been hypothesized that lower socio-economic status can accelerate biological ageing, and predispose to early onset of disease. This study investigated the association of socio-economic and lifestyle factors, as well as traditional and novel risk factors, with biological-ageing, as measured by telomere length, in a Glasgow based cohort that included individuals with extreme socio-economic differences.

**Methods:**

A total of 382 blood samples from the pSoBid study were available for telomere analysis. For each participant, data was available for socio-economic status factors, biochemical parameters and dietary intake. Statistical analyses were undertaken to investigate the association between telomere lengths and these aforementioned parameters.

**Results:**

The rate of age-related telomere attrition was significantly associated with low relative income, housing tenure and poor diet. Notably, telomere length was positively associated with LDL and total cholesterol levels, but inversely correlated to circulating IL-6.

**Conclusions:**

These data suggest lower socio-economic status and poor diet are relevant to accelerated biological ageing. They also suggest potential associations between elevated circulating IL-6, a measure known to predict cardiovascular disease and diabetes with biological ageing. These observations require further study to tease out potential mechanistic links.

## Introduction

Gompertz (1825) first described ageing as an increase in the likelihood of mortality with increasing chronological age [1]. In man, this equates to a corresponding, progressive loss of metabolic and physiological functions, though the trajectory for this is not uniform, indicative of underlying inter-individual variation in the biology of ageing [2].

Variation in the rate of biological ageing reflects the cumulative burden of genetic, metabolic and environmental stressors, resulting in oxidative damage and elevated inflammatory processes [3]. This is of particular interest in Glasgow, because of the exceptional gradient of socio-economic status (SES) in this city and the associated variation in mortality and morbidity. The latter is reflected in the large difference in life expectancy for men, between the most and least deprived areas of the city, which is 28.7 years [4]; a difference which is one of the largest in the developed world. We investigated potential mechanisms for such gradients as part of the psychological, social, and biological determinants of ill health (pSoBid) study cohort [5], the characteristics of which have been described in depth elsewhere [6]. Briefly, the pSoBid study was designed to investigate the factors linking social circumstances, mental wellbeing, and biological markers of disease. Participants were selected from the least and most deprived areas in the NHS Greater Glasgow Health Board area. We have hypothesised that such a difference in life expectancy might be reflected in the biological age of individuals. In turn we considered whether deprivation-related measures correlated to accelerated telomere attrition and also determined interactions with inflammatory status.

Suitable and validated biomarkers for analysing biological ageing in this context are limited in number. Despite cell cycle inhibitor transcript levels providing an accurate indication of organ and T cell biological age [7]–[9] the overwhelming majority of studies employ determination of telomere length in peripheral blood leukocytes (PBLs) in a clinical context [10]–[12]. However, there are many equivocal reports regarding how useful this marker is when applied in epidemiological studies [13], [14].

Telomeres are nucleoprotein complexes at chromosome ends, consisting of (TTAGGG)_n_ direct repeats bound to a range of proteins involved in maintaining cellular stability and viability (reviewed in [3]). Typically, in larger mammals, the telomeric DNA component shortens with successive cell divisions. This arises from what has been termed the end replication problem [15]. Telomere attrition is thought to represent a molecular clock, at least at the cellular level, where it has been hypothesised to act as an anti-neoplastic mechanism [16], proposed to function by countering the accumulation of mutations over successive cell divisions, which potentially could produce neoplastic cells.

Although telomere length is inversely related to chronological age in humans, there is considerable inter-individual variation in telomere length at any specific age [17]. Thus estimates of the effects of chronological age as a covariable in regression models have relatively wide confidence intervals, making detection of significant effects of age-related diseases more difficult: it may be unclear how much of the residual variance is explained by the disease and how much is attributable to error in the estimate of an age effect. This has been discussed in detail eleswhere [18], [19].

Recent human data have now established telomere attrition as a major risk factor for numerous diseases, including cardiovascular disease (CVD), hypertension, diabetes and end stage renal disease [11], [20]–[22] as well as being associated with elevated psychological stresses [23]. Many such pathologies, showing an association with increased telomere attrition rates, are predominant in deprived communities where there is a higher prevalence of classical risk factors for disease, but this explanation does not account totally for these variations in disease incidence [10], [24], [25].

One hypothesis for the increased disease prevalence in these communities is underlying chronic inflammation (elevated CRP or IL-6), a known component and predictor of CVD and diabetes [26], [27], that is linked to a diverse range of pathologies. A possible contributory factor to generating an increased pro-inflammatory state, is accelerated biological ageing. Both telomere attrition and CDKN2A expression have been reported to show association with IL-6 levels in disease [11] and ostensibly ‘healthy’ populations [9]. Such associations are intuitive, as senescent cells upregulate and secrete pro-inflammatory cytokines as part of the senescent secretosome [28].

A link between accelerated biological ageing and socio-economic status has previously been reported by some [29], but not by others [30], [31]. The reasons for this equivocacy remain to be proven, but may be attributable to methodological differences [13]. Any putative link, however, may be weak and open to multiple confounders, such as parental telomere length and epigenetic effects [32].

We have chosen to evaluate the contribution of socio-economic factors to biological age, as measured by telomere length, in the extreme setting of the pSoBid cohort, to determine to what extent this in turn affects risk factors for ill health.

## Results

### Socio-economic and lifestyle factors

Telomere lengths were determined in PBLs by Q-PCR for 382 individuals. The SES and lifestyle factors investigated are shown in Table S1, as are the median telomere lengths within subgroups of these factors, overall and by three age groups.

The relationship between age, gender and biological ageing was investigated by estimating the percentage change in telomere length associated with a decade increase in age and male gender. Age was strongly negatively associated with telomere length, each decade predicting a 4.8% decrease in telomere length (p = 0.002). No significant difference in telomere length was observed between males and females. There was also no difference between affluent and deprived groups either in telomere length or age-related telomere attrition. All subsequent analyses were adjusted for age, gender and deprivation.

**Figure 1:**
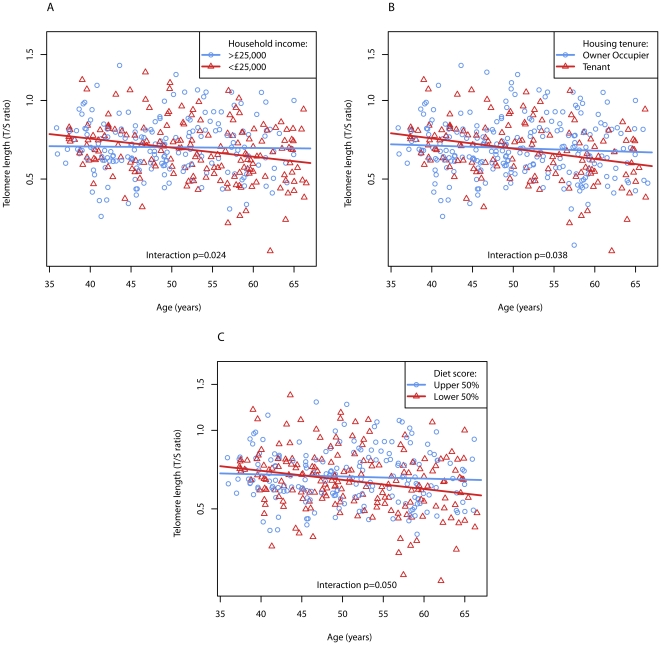
Regression analysis plots that highlight household income, housing tenure, and diet score were associated with steeper age-related decline in telomere length. Faster rates of age-related telomere attrition were observed in individuals with an average income less than £25,000 (7.7% vs 0.6% reduction per decade, p = 0.024, (A)), home tenants (8.7% vs 2.2%, p = 0.038 (B)) and a diet score among the lower 50% of scores (7.7% vs 1.8%, p = 0.05 (C)).

Of the SES and lifestyle factors investigated, only cigarette smoking was associated with an overall reduction in telomere length (6.6% reduction, p = 0.050). However, household income, housing tenure, and diet score were associated with steeper age-related decline in telomere length (Table 1). Faster rates of age-related telomere attrition were observed in individuals with an average income less than £25,000 (7.7% vs 0.6% reduction per decade, p = 0.024), a diet score among the lower 50% of scores (7.7% vs 1.8%, p = 0.05), and home tenants (8.7% vs 2.2%, p = 0.038). Figure 1 highlights differences in telomere attrition with income, housing tenure and diet score.

**Table 1:** Highlights the percentage change in telomere length per decade that is associated with SES and lifestyle factors.

	% change in telomere length (95% CI) per decade associated with SES & lifestyle factor		
SES & Lifestyle Factor	SES & Lifestyle Factor Subgroup NO	SES & Lifestyle Factor Subgroup YES	Differences between subgroups (p-value)
**Social class: Manual**	−3.7 (−7.5, 0.3) p = 0.066	−6.2 (−11.1, −1.1) p = 0.019	0.437
**Household income: < £25,000**	−0.6 (−5.3, 4.2) p = 0.796	**−7.7 (−11.6, −3.6) p<0.001**	**0.024**
**Years of education: Lower 50%**	−1.3 (−5.9, 3.5) p = 0.577	−7.2 (−11.0, −3.2) p = 0.001	0.062
**Housing tenure: Tenant**	−2.2 (−6.1, 1.8) p = 0.274	**−8.7 (−13.1, −4.0) p<0.001**	**0.038**
**Physical activity level: Inactive**	−6.0 (−10.5, −1.3) p = 0.013	−3.6 (−7.5, 0.6) p = 0.092	0.426
**Current cigarette smoker**	−4.8 (−8.2, −1.3) p = 0.008	−7.4 (−13.6, −0.8) p = 0.028	0.482
**Diet score: Lower 50%**	−1.8 (−6.1, 2.6) p = 0.417	**−7.7 (−11.7, −3.6) p<0.001**	**0.050**
**Excessive alcohol (>14 [F] or 21 [M] U/week)**	−4.8 (−8.0, −1.3) p = 0.007	−5.0 (−11.6, 2.0) p = 0.156	0.944
**Obese (BMI >30 kg/m2)**	−4.1 (−7.5, −0.4) p = 0.029	−7.2 (−12.8, −1.2) p = 0.019	0.371
**Waist/Hip ratio: Upper 50%**	−2.2 (−6.5, 2.2) p = 0.319	−7.6 (−11.6, −3.4) p = 0.001	0.081

Individuals with an income < £25,000, housing tenants and those with a lower diet score were shown to have faster rates of age-related telomere attrition than those earning >£25,000, house owners and individuals with higher diet scores. All analyses were adjusted for age, gender and deprivation group.

We investigated the extent to which the associations between telomere attrition and household income, housing tenure, and diet score were independent by adjusting for these interactions. All three interactions were attenuated to a similar degree and were no longer significant, suggesting that these three interactions are correlated but no single factor is driving these interactions (Table S2).

### Biomarkers

Associations between biomarkers investigated and telomere length are reported in Table 2. Individuals with longer telomeres had increased levels of total cholesterol (2.4% increase in total cholesterol per one SD increase in log telomere length, p = 0.027) and LDL cholesterol (3.7%, p = 0.027). Conversely, shorter telomeres were associated with increased levels of IL-6 (7.2% decrease in IL6 levels per one SD increase in log telomere length, p = 0.022, Table 2). Further analysis, adjusting for deprivation, income, diet and smoking status, maintained the positive association between telomere length and cholesterol (p = 0.033) and LDL cholesterol (p = 0.026). Interestingly, the significant association between telomere length and IL-6 was weakened when adjusted for these SES factors. Analyses were performed to establish which individual factors had an impact on IL-6 expression (Table 3). The significant association between shorter telomeres and higher levels of IL-6 was lost when analyses were adjusted for age and gender, or for smoking status deprivation, income and diet (Table 3).

**Table 2:** Highlights the percentage change in biomarker levels that is associated with an increase in telomere length.

Outcome	Percentage Change (95% CI)p-Value
Systolic BP (mmHg)	0.2 (−1.1, 1.5) p = 0.798
Diastolic BP (mmHg)	0.3 (−1.0, 1.7) p = 0.642
Cholesterol (mmol/l)	2.4 (0.3, 4.6) p = 0.027
HDL cholesterol (mmol/l)	−1.2 (−3.9, 1.6) p = 0.387
LDL cholesterol (mmol/l)	**3.7 (0.4, 7.1) p = 0.027**
Triglycerides (mmol/l)	2.7 (−2.6, 8.3) p = 0.326
Glucose (mmol/l)	0.8 (−1.0, 2.7) p = 0.363
Insulin (mU/l)	−0.5 (−7.5, 6.9) p = 0.884
HOMA - IR	−0.1 (−7.5, 8.0) p = 0.989
C reactive protein (mg/l)	0.6 (−9.9, 12.4) p = 0.916
Interleukin 6 (pg/ml)	**−7.2 (−12.9, −1.1) p = 0.022**
Intercellular adhesion molecule 1 (ng/ml)	−0.3 (−2.9, 2.3) p = 0.796
Fibrinogen (g/l)	0.2 (−1.9, 2.4) p = 0.829
von Willebrand factor (IU/dl)	−1.2 (−4.2, 1.8) p = 0.416
D-dimer (ng/ml)	−1.7 (−7.4, 4.4) p = 0.583

Individuals with longer telomeres had increased levels of total cholesterol (2.4% increase, p = 0.027) and LDL cholesterol (3.7%, p = 0.027). Conversely, shorter telomeres were associated with increased levels of IL-6 (7.2% decrease, p = 0.022). All analyses were adjusted for age, gender and deprivation group. The change in telomere length was measured by an increase of one standard deviation in log telomere length.

**Table 3:** Highlights the percentage change in IL6 levels that is associated with an increase in telomere length.

Adjustment covariates	% Change in IL6 Levels (95% CI)
None	−9.9 (−16.3, −3.0), p = 0.006
Age + gender	−7.0 (−13.6, 0.1), p = 0.052
… + current smoking	−5.9 (−12.4, 1.0), p = 0.094
… + deprivation group	−5.7 (−11.9, 0.9), p = 0.090
… + income ≥/< £25,000	−5.6 (−11.8, 1.0), p = 0.093
… + diet score ≥/< median	−5.6 (−11.8, 1.0), p = 0.096

This analysis was adjusted for the various different SES and lifestyle factors to determine their contribution to the difference in IL6 levels. The change in telomere length was measured by an increase of one standard deviation in log telomere length.

## Discussion

It has been hypothesized that socio-economic deprivation can accelerate biological ageing, resulting in shorter telomeres in deprived individuals in comparison to more affluent-aged matched controls. Five previous studies examining this relationship report positive [29], null [30], [33], [34] and negative associations [31]. The equivocacy between these reports is possibly due to methodological differences, variations inherent in individual cohorts and in the veracity of subject answers relating to SES data, such as income, which may be confounded by undeclared income. The present study has examined the relationship between biological ageing, SES and disease in participants in Glasgow, a city with an extreme socioeconomic gradient, with documented health issues associated with social deprivation.

Interestingly, in the light of possible confounders relating to the veracity of SES data, employment status (men who reported being out of work) was reported to associate significantly with shorter telomeres [34], in a large cross sectional study from an overlapping demographic area (the WOSCOPS cohort). Surprisingly, despite the prevalence of CVD in this cohort and its proven association with SES, there were no other associations found with other markers of SES (including educational attainment, employment status, area-based deprivation and physical stature measured as a proxy for early life social circumstances).

Our data are not incongrous with previous reports, as we observed no associations with area based deprivation and employment. However, we have demonstrated a direct link between accelerated biological ageing, low income and poor diet. Furthermore, we have observed a relationship with a measure of adiposity, namely waist/hip ratio (Table 1), a predictive measure for CVD and diabetes as well as all cause mortality in prospective studies [35].

These observations are intuitive and in keeping with the Marmot findings [36], [37] who indicated that relative health inequalities are associated with SES. It is reasonable to assume that a low relative income means a decreased likelihood of being able to afford a good quality diet, leading to an acceleration in biological ageing. Poorer quality, fat and sugar rich diets, are known to result in the production of more reactive oxygen species, which directly cause DNA breaks that lead to gene malfunction, telomere attrition and disease [38], [39].

Notably, these observations indicating an interaction between biological ageing and SES are reinforced by the finding that telomere length, in the pSoBid cohort, associates positively with LDL cholesterol levels, a strong and unambiguous causal risk factor in CVD.

In our study, telomere attrition was associated with increasing IL-6 levels, an emerging risk factor for CVD, which may predict fatal events more strongly than non-fatal events [40]. The association of IL-6 with biological age is in keeping with recent observations indicating that senescent cells up-regulate and secrete IL-6 [9], [11]. It would be expected, as a consequence of increased telomere attrition, that this group would have more senescent cells present and thus higher IL-6 levels and have an elevated risk of a range of conditions, if indeed IL-6 is causally related to CVD and diabetes. The association between accelerated biological ageing and increased IL-6 levels has been previously been reported to be linked with disease [11], general health [9] and social deprivation [41]. These observations are congruent with more extant inflammatory conditions in the most deprived, a situation exacerbated by increasing age.

Our findings suggest the unadjusted association between telomere length and IL-6 is strong, and it is still marginally significant with adjustment for age and gender. This association is only partially weakened by further adjusting for smoking but subsequent addition of deprivation, income and diet does not appear to weaken the IL-6-telomere association further (Table 3). In simple terms, these observations suggest that varying upstream factors drive both telomere shortening and elevated IL-6 levels. However, we cannot exclude the possibility that part of the mechanism for elevated IL-6 is via telomere shortening. Future studies need to explore this potential further, particularly as IL-6 is attracting increasing interest in the diabetes and CVD arenas [27], [40].

Of the four previous studies in this field [29]–[31], [34] only two have indicated an association with SES. These comprised an analysis of female twins, where non manual workers had longer telomere lengths than manual workers [29]. However, in analyses in which the authors used a more comprehensive range of SES categories, no evidence of a relationship was observed with biological age. The other, analysed Chinese men and indicated men with higher self-rated socioeconomic status have shorter telomeres [31]. This was postulated as possibly being mediated through psychosocial, rather than lifestyle factors, or the presence of chronic disease. These authors also argue that there may be significant cultural, ethnic and age-related differences in social determinants of health.

The Q-PCR methodology employed in the present study yielded similar telomere length data in keeping with other reports [10], [33], [42] using the same methodology. Our observations must also be viewed with reference to the fact that any differences reflect that PBL telomere length is neither an absolute, nor precise measure of biological ageing. The telomere lengths measured are an average and reflect a range of lengths in cells. A better measure may be an absolute marker of cellular growth arrest, such as CDKN2A [7]–[9]. The use of such a marker would avoid the potentially confounding effects of differences in telomere length measurement methodology, that currently beset the field [14].

The difference in observations using telomere length as a marker of biological ageing, that have been reported by different groups have been elegantly summarised by Nordfjall et al [39]. Our observations find consensus with those using a similar methodology, when applied to age, BMI, smoking, insulin, triglycerides and glucose. Our observations on the effect of smoking, however, maybe limited by lack of detailed information on smoking history.

We differ in the detections of associations with total and LDL cholesterol, though these have previously been reported to be associated with telomere length in a disease setting [11], along with IL6 [7], [9]. These observations were also in keeping with concomitant elevation of DNA damage (as measured by 8-0H dG levels) in a specific disease cohort. However, our observations are consistent with previous biochemical analysis of the pSoBid cohort [5], which demonstrated that total cholesterol and LDL were inversely associated with deprivation. This is congruent with more extant inflammatory conditions in the most deprived, a situation exacerbated by increasing age. It is thus also in keeping with the observed elevation of IL-6 correlating with short telomeres.

Our observations provide an intuitive link between proven socio-economic drivers of disease [5], [36], [37] and a biological phenomenon underlying any predisposition, or extant disease, namely cellular ageing. The tendency of our data to show faster ageing in those with lower socio-economic status, is indicative of this and reflects ‘more miles on the clock’ for these individuals.

This study may be limited by its size and cross sectional nature. Indeed, the social gradient in Glasgow is so extreme that a ‘survivor effect’ among the most deprived cannot be excluded. This merits a larger, longitudinal study to look at the relative impacts of further markers of SES and potential SE and lifestyle interventions. Such interventions are not without precedence and appear to show direct benefit to biological ageing. A recent intervention study in men with prostate cancer, reported that changing lifestyle, primarily via better diet and increased exercise leads to increased telomerase activity and deceleration of telomere attrition rate [43]. A similar result in the pSoBid cohort would be expected to have significant health benefits.

In summary, we show convincingly that factors associated with lower socio-economic status and poor diet are relevant to accelerated biological ageing in a cohort representing extremes of social class. Our findings also suggest potential associations of elevated circulating IL-6, a measure known to predict CVD and diabetes, with biological ageing, observations which require further study to tease out potential mechanistic links.

## Materials and Methods

### Ethics Statement

The study was approved by the Glasgow Royal Infirmary Research Ethics Committee and all participants gave written informed consent.

### Participants

The design of the psychological, social, and biological determinants of ill health (pSoBid) study has been described elsewhere [6]. In brief, participants were ranked on the basis of multiple deprivation indicators to define the least and most deprived areas in the NHS Greater Glasgow Health Board area, using criteria established in the Scottish Index for Multiple Deprivation (SIMD). Sampling was stratified to achieve an approximately equal distribution of the 666 participants across males and females and age groups (35–44, 45–54 and 55–64 years) within the most (bottom 5% of SIMD score) and least deprived areas (top 20% of SIMD score). Participants undertook a physical examination (including measurement of blood pressure, body mass index (BMI), and waist hip ratio (WHR)) and lifestyle questionnaires as detailed previously [6]. A total of 382 blood samples were available for telomere analysis.

### Analytical parameters

Measurement of biochemical parameters have been described in detail elsewhere [5]. In brief, cholesterol and triglyceride concentrations were determined by enzymatic colorimetric assays on a Roche Hitachi 917 analyser (Roche Diagnostics Ltd, Burgess Hill, UK). Lipid fractions were measured using ultracentrifugation and precipitation methods. Glucose was measured by hexokinase and glucose-6-phosphate dehydrogenase assay on an Abbott c8000 analyser (Abbott Diagnostics, Maidenhead, UK). Insulin was measured by ELISA (Mercodia AB, Uppsala, Sweden and ALPCO Diagnostics, Salem, NH, USA, respectively). C-reactive protein levels were determined by an immunoturbidimetric assay (Roche Diagnostics Ltd). High sensitivty interleukin-6, von Willebrand factor and intercellular adhesion molecule 1 were measured by ELISA (R&D Systems Europe Ltd, Abingdon, UK and DAKO UK Ltd, Ely, UK). Fibrinogen was measured on an automated coagulometer (MDA-180; Organon Teknika, Cambridge, UK). D-dimer was measured by ELISA (Hyphen, Neuville-sur-Oise, France).

### Indices of dietary intake

A diet score for the consumption of fruit and vegetables was calculated from subjects self-reported food frequency questionnaire responses. Participants were asked on average how often they consumed a range of food categories (21 food categories listed). Responses for each question ranged from daily consumption (number of portions per day) to weekly and monthly consumption. Participants selected one response per food category. For the purposes of the present analysis, responses to four questions from the food frequency questionnaire relating to fruit and vegetable intake were aggregated to give an overall indicative diet score (i.e. frequency of intake of fresh fruit, cooked green vegetables (fresh or frozen), cooked root vegetables (fresh or frozen) and raw vegetables or salad (including tomatoes)). Monthly diet scores were calculated on the basis of a 28 day month. The maximum possible total diet score was 672 (6 portions per day x 28 days per month x 4 food category questions).

### Telomere length determination

DNA was extracted from PBLs following standard procedures and telomere lengths in the DNA samples were determined by Q-PCR, following the method of Cawthon [44] as described previously [11], [33]. Telomere length determination was performed using a Roche Light Cycler LC480. Telomere length analyses were performed in triplicate for each sample, using a single-copy gene amplicon primer set (acidic ribosomal phosphoprotein, 36B4) and a telomere-specific amplicon primer set [7]. Quality control parameters employed for the amplifications comprised using a cut off 0.15 for the standard deviation (SD) of the threshold cycle (Ct) for sample replicates. At a SD above 0.15 the sample was reanalysed. The average SD across plates was 0.05.

Relative telomere length was estimated from Ct scores using the comparative Ct method after confirming that the telomere and control gene assays yielded similar amplification efficiencies. This method determines the ratio of telomere repeat copy number to single copy gene number (T/S) ratio in experimental samples relative to a control sample DNA. This normalised T/S ratio was used as the estimate of relative telomere length (Relative T/S).

The inter-assay variation was assessed by comparing the relative telomere estimates (T/S ratio) across assays for the positive controls, which were assayed on every assay plate. The average inter-assay coefficient of variance was 0.3% for telomere and 0.1% for 36B4 plates.

### Statistical analyses

Associations between telomere length and participant characteristics were investigated in linear regression models. Sampling was stratified by age, gender and deprivation group, and all models were adjusted for these factors. Because telomeres are expected to shorten gradually with age, age was represented in models as a continuous rather than categorical covariate.

When investigating factors that might influence ageing such as SES and lifestyle, telomere length was modelled as an outcome. Biomarkers, on the other hand, may be viewed as downstream of ageing, motivating their modelling as outcomes with telomere length as a covariate.

Telomere length and biomarkers were log-transformed for regression analysis to satisfy the assumption of normally distributed residuals. Regression coefficient estimates were therefore multiplicative when transformed back to the original scale. For example, a regression coefficient for a binary characteristic back-transormed to 1.1 implies the characteristic is associated with a 10% difference in the outcome. Thus, where log telomere length was the outcome, regression coefficients are presented as the percentage change in telomere length associated with each patient characteristic. Where telomere length was a covariate, a telomere length z-score was used so that the back-transformed regression coefficients could be interpreted as the percentage change in biomarker level associated with a one standard deviation increase in telomere length. The telomere length z-score was calculated by standardising log telomere length to have a mean of zero and standard deviation of one.

We hypothesised that the effects of telomere-shortening factors accumulate over time, therefore the largest differences between exposed and unexposed participants would be expected among the oldest participants. We investigated this hypothesis by testing for interactions between participant characteristics and age.

## Supporting Information

Table S1 **Median (interquartile range) telomere length within subgroups of socioeconomic status and lifestyle factors, overall and by age group.**
(DOC)

Table S2 **Percentage change (95% CI) in telomere length associated with a 10-year increase in age within subgroups of socioeconomic status and lifestyle factors, predicted from linear regression models adjusted for significant main effects and interactions.** All models were adjusted for age, gender and deprivation group. Interactions were investigated by testing the null hypothesis of homogeneity of age effects across subgroups.
(DOC)
