# Supplementary material for: Accelerated Telomere Attrition Is Associated with Relative Household Income, Diet and Inflammation in the pSoBid Cohort
Source: PLoS One. 2011 Jul 27;6(7):e22521. doi: 10.1371/journal.pone.0022521 (PMC3144896; doi:10.1371/journal.pone.0022521)
Supplement: Table S1 — Median (interquartile range) telomere length within subgroups of socioeconomic status and lifestyle factors, overall and by age group. (DOC) [file pone.0022521.s001.doc]

| Characteristic | Subgroup | All | Age group | | |
| --- | --- | --- | --- | --- | --- |
|  |  |  | 35-44 | 45-54 | 55-64 |
| Deprivation group | Affluent Deprived | 0.65 (0.56, 0.77) 0.65 (0.55, 0.77) | 0.66 (0.59, 0.78) 0.69 (0.60, 0.77) | 0.64 (0.57, 0.76) 0.67 (0.55, 0.79) | 0.64 (0.52, 0.77) 0.59 (0.49, 0.70) |
| Social class | Non-Manual Manual | 0.65 (0.56, 0.77) 0.64 (0.55, 0.75) | 0.68 (0.60, 0.78) 0.67 (0.60, 0.76) | 0.66 (0.57, 0.77) 0.63 (0.55, 0.77) | 0.62 (0.52, 0.76) 0.61 (0.49, 0.71) |
| Household income | > £25,000 < £25,000 | 0.65 (0.58, 0.77) 0.65 (0.55, 0.76) | 0.65 (0.59, 0.78) 0.70 (0.61, 0.77) | 0.64 (0.57, 0.76) 0.67 (0.56, 0.79) | 0.65 (0.53, 0.77) 0.59 (0.49, 0.71) |
| Years of education | Upper 50% Lower 50% | 0.64 (0.55, 0.78) 0.65 (0.55, 0.75) | 0.68 (0.60, 0.78) 0.67 (0.60, 0.77) | 0.63 (0.56, 0.77) 0.67 (0.59, 0.76) | 0.64 (0.52, 0.78) 0.60 (0.49, 0.70) |
| Housing tenure | Owner Occupier Tenant | 0.64 (0.56, 0.77) 0.66 (0.55, 0.76) | 0.65 (0.60, 0.78) 0.71 (0.61, 0.77) | 0.64 (0.57, 0.76) 0.69 (0.55, 0.80) | 0.63 (0.51, 0.77) 0.58 (0.50, 0.70) |
| Physical activity level | Active Inactive | 0.65 (0.56, 0.78) 0.65 (0.54, 0.76) | 0.67 (0.59, 0.79) 0.69 (0.61, 0.77) | 0.68 (0.59, 0.80) 0.61 (0.54, 0.73) | 0.57 (0.50, 0.71) 0.63 (0.51, 0.76) |
| Current cigarette smoker | No Yes | 0.65 (0.56, 0.77) 0.64 (0.54, 0.73) | 0.67 (0.60, 0.78) 0.68 (0.62, 0.74) | 0.68 (0.58, 0.77) 0.60 (0.53, 0.76) | 0.61 (0.50, 0.75) 0.60 (0.49, 0.66) |
| Diet score | Upper 50% Lower 50% | 0.65 (0.57, 0.77) 0.64 (0.54, 0.75) | 0.65 (0.60, 0.78) 0.70 (0.59, 0.77) | 0.66 (0.56, 0.78) 0.64 (0.56, 0.76) | 0.65 (0.53, 0.77) 0.59 (0.48, 0.70) |
| Excessive alcohol (> 14 [F] or 21 [M] U/week) | No Yes | 0.65 (0.55, 0.77) 0.64 (0.56, 0.76) | 0.67 (0.59, 0.78) 0.69 (0.63, 0.76) | 0.67 (0.57, 0.77) 0.63 (0.56, 0.70) | 0.62 (0.50, 0.74) 0.60 (0.52, 0.76) |
| Obese (BMI >30 kg/m^2^) | No Yes | 0.64 (0.55, 0.77) 0.66 (0.55, 0.77) | 0.67 (0.59, 0.77) 0.71 (0.63, 0.78) | 0.63 (0.56, 0.77) 0.68 (0.58, 0.76) | 0.61 (0.51, 0.75) 0.62 (0.48, 0.74) |
| Waist/Hip ratio | Lower 50% Upper 50% | 0.64 (0.55, 0.76) 0.66 (0.55, 0.77) | 0.65 (0.59, 0.74) 0.73 (0.62, 0.78) | 0.63 (0.56, 0.77) 0.67 (0.55, 0.75) | 0.61 (0.51, 0.75) 0.60 (0.49, 0.74) |

**Table S1**
