# Supplementary material for: Accelerated Telomere Attrition Is Associated with Relative Household Income, Diet and Inflammation in the pSoBid Cohort
Source: PLoS One. 2011 Jul 27;6(7):e22521. doi: 10.1371/journal.pone.0022521 (PMC3144896; doi:10.1371/journal.pone.0022521)
Supplement: Table S2 — Percentage change (95% CI) in telomere length associated with a 10-year increase in age within subgroups of socioeconomic status and lifestyle factors, predicted from linear regression models adjusted for significant main effects and interactions. All models were adjusted for age, gender and deprivation group. Interactions were investigated by testing the null hypothesis of homogeneity of age effects across subgroups. (DOC) [file pone.0022521.s002.doc]

| Predictor | Subgroup |  | Adjusted for household income and age interaction | |  | Adjusted for tenure and age interaction | |  | Adjusted for diet score and age interaction | |  | Adjusted for current smoking | |  | Adjusted for waist/hip ratio and age interaction | | | | |
| --- | --- | --- | --- | --- | --- | --- | --- | --- | --- | --- | --- | --- | --- | --- | --- | --- | --- | --- | --- |
|  |  |  | Age effect (per decade) | Interact- ion p-value |  | Age effect (per decade) | Interact- ion p-value |  | Age effect (per decade) | Interact- ion p-value |  | Age effect (per decade) | Interact- ion p-value |  | | Age effect (per decade) | Interact- ion p-value | |  |
| Social class | Non-Manual Manual |  | -0.7  (-5.5, 4.4) -0.5  (-8.2, 7.8) | 0.968 |  | -2.3  (-6.4, 2.0) -1.5  (-8.6, 6.2) | 0.824 |  | -1.4  (-6.1, 3.4) -2.7  (-9.2, 4.4) | 0.721 |  | -4.2  (-8.0, -0.3) -6.7  (-11.8, -1.5) | 0.446 |  | -1.8  (-6.4, 3.1) -3.8  (-10.2, 3.2) | | | 0.561 | |
| Household income | > £25,000 < £25,000 |  | -  - | - |  | -0.4  (-5.1, 4.5) -4.6  (-11.7, 3.1) | 0.342 |  | 0.8  (-4.4, 6.3) -5.1  (-10.6,  0.7) | 0.075 |  | -0.7  (-5.4,  4.2) -8.7  (-12.7, -4.5) | 0.012 |  | 1.0  (-4.2, 6.6) -5.7  (-11.1, 0.0) | | | 0.042 | |
| Years of education | Upper 50% Lower 50% |  | 0.2  (-4.8, 5.4) -3.4  (-10.6, 4.4) | 0.369 |  | -0.7  (-5.4, 4.2) -4.3  (-10.0, 1.8) | 0.334 |  | 0.6  (-4.6, 6.1) -4.2  (-9.5, 1.4) | 0.144 |  | -2.2  (-6.8,  2.7) -7.7  (-11.6, -3.6) | 0.082 |  | 0.5  (-4.9, 6.1) -5.0  (-10.1, 0.5) | | | 0.094 | |
| Housing tenure | Owner Occupier Tenant |  | -0.4  (-5.1, 4.5) -4.9  (-13.6, 4.8) | 0.310 |  | -  - | - |  | -0.4  (-5.0, 4.5) -5.7  (-11.7,  0.7) | 0.106 |  | -3.0  (-6.9,  1.0) -9.1  (-13.6, -4.2) | 0.057 |  | -0.2  (-5.0,  4.8) -6.6  (-12.4, -0.5) | | | 0.047 | |
| Physical activity level | Active Inactive |  | -2.7  (-8.1, 3.1)  2.0  (-3.8, 8.2) | 0.159 |  | -4.3  (-9.1, 0.8)  0.4  (-4.9, 5.9) | 0.164 |  | -3.5  (-8.6, 1.9)  0.5  (-5.0, 6.4) | 0.222 |  | -7.0  (-11.5, -2.3) -4.0  (-8.1,  0.3) | 0.342 |  | -3.4  (-8.9, 2.3) -1.0 (-6.2, 4.4) | | | 0.455 | |
| Current cigarette smoker | No Yes |  | -0.9  (-5.6,  4.1)  1.3  (-7.4, 10.8) | 0.604 |  | -3.0 (-7.0, 1.1) -2.9  (-11.0, 6.0) | 0.965 |  | -2.4  (-6.8, 2.2) -3.2  (-11.0,  5.2) | 0.832 |  | -  - | - |  | -1.1  (-5.7, 3.8) -3.7  (-10.9, 4.0) | | | 0.498 | |
| Diet score | Upper 50% Lower 50% |  | 0.8  (-4.4, 6.3) -3.5  (-9.5, 2.9) | 0.186 |  | -0.4  (-5.0, 4.5) -5.0  (-10.2, 0.4) | 0.145 |  | -  - | - |  | -2.5  (-6.8,  2.0) -8.3  (-12.4, -4.1) | 0.058 |  | 0.4  (-4.8,  5.8) -5.7  (-10.9, -0.2) | | | 0.053 | |
| Excessive alcohol (> 14 [F] or 21 [M] U/week) | No Yes |  | -0.6  (-5.5, 4.6) -1.1  (-8.5, 7.0) | 0.908 |  | -2.2  (-6.4, 2.1) -2.5  (-9.6, 5.2) | 0.952 |  | -2.0  (-6.3, 2.6) -0.7  (-8.6, 7.9) | 0.750 |  | -4.8  (-8.1, -1.3) -8.1  (-14.9, -0.7) | 0.417 |  | -2.1  (-6.6, 2.6) -2.3  (-9.7, 5.7) | | | 0.961 | |
| Obese (BMI >30 kg/m^2^) | No Yes |  | -0.5  (-5.3, 4.6) -1.1  (-8.4, 6.6) | 0.856 |  | -1.6  (-5.8, 2.9) -4.4  (-10.7, 2.4) | 0.434 |  | -0.9  (-5.6, 4.1) -4.3  (-10.6,  2.5) | 0.346 |  | -4.1  (-7.6, -0.4) -8.8  (-14.4, -2.8) | 0.179 |  | -2.1  (-6.5, 2.5) -3.2  (-11.1, 5.4) | | | 0.777 | |
| Waist/Hip ratio | Lower 50% Upper 50% |  | 1.0  (-4.2, 6.6) -3.1  (-9.0, 3.2) | 0.205 |  | -0.2  (-5.0, 4.8) -4.6  (-9.6, 0.8) | 0.170 |  | 0.4  (-4.8, 5.8) -4.2  (-9.5, 1.5) | 0.150 |  | -1.6  (-6.0,  3.0) -9.1  (-13.1, -4.8) | 0.016 |  | -  - | | | - | |

**Table S2**
